# Why Birds with Deferred Sexual Maturity Are Sedentary on Islands: A Systematic Review

**DOI:** 10.1371/journal.pone.0022056

**Published:** 2011-07-19

**Authors:** Miguel Ferrer, Keith Bildstein, Vincenzo Penteriani, Eva Casado, Manuela de Lucas

**Affiliations:** 1 Department of Ethology and Biodiversity Conservation, Estación Biológica de Doñana, Consejo Superior de Investigaciones Cientificas (CSIC), Seville, Spain; 2 Acopian Center for Conservation Learning, Hawk Mountain Sanctuary, Orwigsburg, Pennsylvania, United States of America; 3 Department of Conservation Biology, Estación Biológica de Doñana, Consejo Superior de Investigaciones Cientificas (CSIC), Seville, Spain; 4 Fundación Migres, Seville, Spain; University of Western Ontario, Canada

## Abstract

**Background:**

Island faunas have played central roles in the development of evolutionary biology and ecology. Birds are among the most studied organisms on islands, in part because of their dispersal powers linked to migration. Even so, we lack of information about differences in the movement ecology of island versus mainland populations of birds.

**Methodology/Principal Findings:**

Here we present a new general pattern indicating that large birds with deferred sexual maturity are sedentary on islands, and that they become so even when they are migratory on the mainland. Density-dependent variation in the age at first breeding affects the survivorship of insular populations and this, in turn, affects the movement ecology of large birds. Because density-dependent variation in the age of first breeding is critical to the long-term survival of small isolated populations of long-lived species, migratory forms can successfully colonize islands only if they become sedentary once there. Analyses of the movement ecology of continental and insular populations of 314 species of raptors, 113 species of Ciconiiformes and 136 species of passerines, along with individual-based population simulations confirm this prediction.

**Conclusions:**

This finding has several consequences for speciation, colonization and survival of small isolated population of species with deferred sexual maturity.

## Introduction

Studies of island faunas have played critical roles in the development of evolutionary biology and ecology since the time of Darwin [1] and Wallace [2]. MacArthur and Wilson's theory of island biogeography [3], which revolutionized the field of biogeography, has served as a starting point for modeling colonization and extinction events in the emerging field conservation biology [4], as well as for discussions regarding how dispersal ability and competition influence the composition of insular faunas [5]–[8]. Although birds are among the most studied organisms on islands, in part because of their dispersal powers linked to migration we lack of information about possible differences in the movement ecology of island versus mainland populations of birds and the extent to which such differences might reflect the situations faced by insular versus continental birds.

Recently, several studies [9]–[12] showed that density-dependent variation in the age of first breeding is critical to the long-term survival of small isolated populations of long-lived species with deferred sexual maturity. This is because density-dependent variation in the age of first breeding buffers population fluctuations and, consequently, increases the persistence of these populations. This buffering effect is such that at low densities, individuals tend to reproduce at younger ages, whereas at higher densities the average age of first breeding increases. Variability in the age of entry into the breeding cohort enables populations to remain closer to carrying capacity, significantly affecting population persistence [10]. The effects of variability in age of first breeding depend on the size of the population, being greater in small than in large ones. This helps to explain the persistence of very small populations of long-lived birds with deferred sexual maturity and density-dependent age of first breeding.

For this mechanism to work, young birds that are able to enter the breeding population need to be able to assess the degree to which that population is saturated so that they can search for and fill vacancies as soon as they become available. This is true for young birds within sedentary populations [13]–[15], which continually move among peripheral nursery areas and central breeding sites until they find a breeding vacancy and enter the reproductive cohort [14]. On the other hand, it has not been observed for young birds in migratory populations, many of which do not return to their breeding areas until the end of a period of delayed sexual maturation [16]–[20]. In this so-called “delayed return” juvenile birds do not participate in two full-distance migrations in the course of the year. In a number of species, sexual maturity is reached only after several years. Juveniles of these species (e.g. raptors, storks and herons) stay on their wintering grounds beyond their first year of life, possibly to avoid unnecessary migration [20] and competition with older birds there. By the time sexual maturity has been attained, these birds will have made their way to the breeding grounds, and from that time on will show normal, complete, periodic twice-annual migrations. Due to this delayed return, sub-adults of these species do not have information about the extent of breeding opportunities on their eventual breeding grounds until they return in adult breeding plumage, and may miss opportunities to breed earlier when vacancies on the breeding grounds exist. Delayed return consequently, is a definitive restraint to density-dependent variation in the age at first breeding.

Here we present a novel explanation on how density-dependence variation in the age at first breeding shapes the movement ecology of large birds on islands and assess the likelihood of their survival on islands as a consequence of this aspect of their ecology. A lack of density-dependent variation in the age at first breeding in long-lived migratory species would have several important consequences [10], one of the most severe being the greater likelihood of extirpation of small isolated populations than occurs in sedentary species with similar demographic parameters. Because of this, we predict that (a) long-lived migratory species, with deferred sexual maturity and delayed return cannot survive for long periods in small isolated populations, typically on islands. Consequently, the only migratory species that will exist in small islands tend to be those without deferred sexual maturity. (b) Migratory populations of long-lived species with deferred sexual maturity that have been able to colonize islands must be sedentary on islands, even if they are migratory on the most proximate continental populations.

## Results

A total of 563 species of birds belonging to three orders (Ciconiiformes and Accipitriformes, both with deferred sexual maturity and retarded-return) and some families of Passeriformes (in which deferred maturity is rarer [21]), were studied, 383 (68%) of these species are sedentary and 180 are migratory.

**Figure 1:**
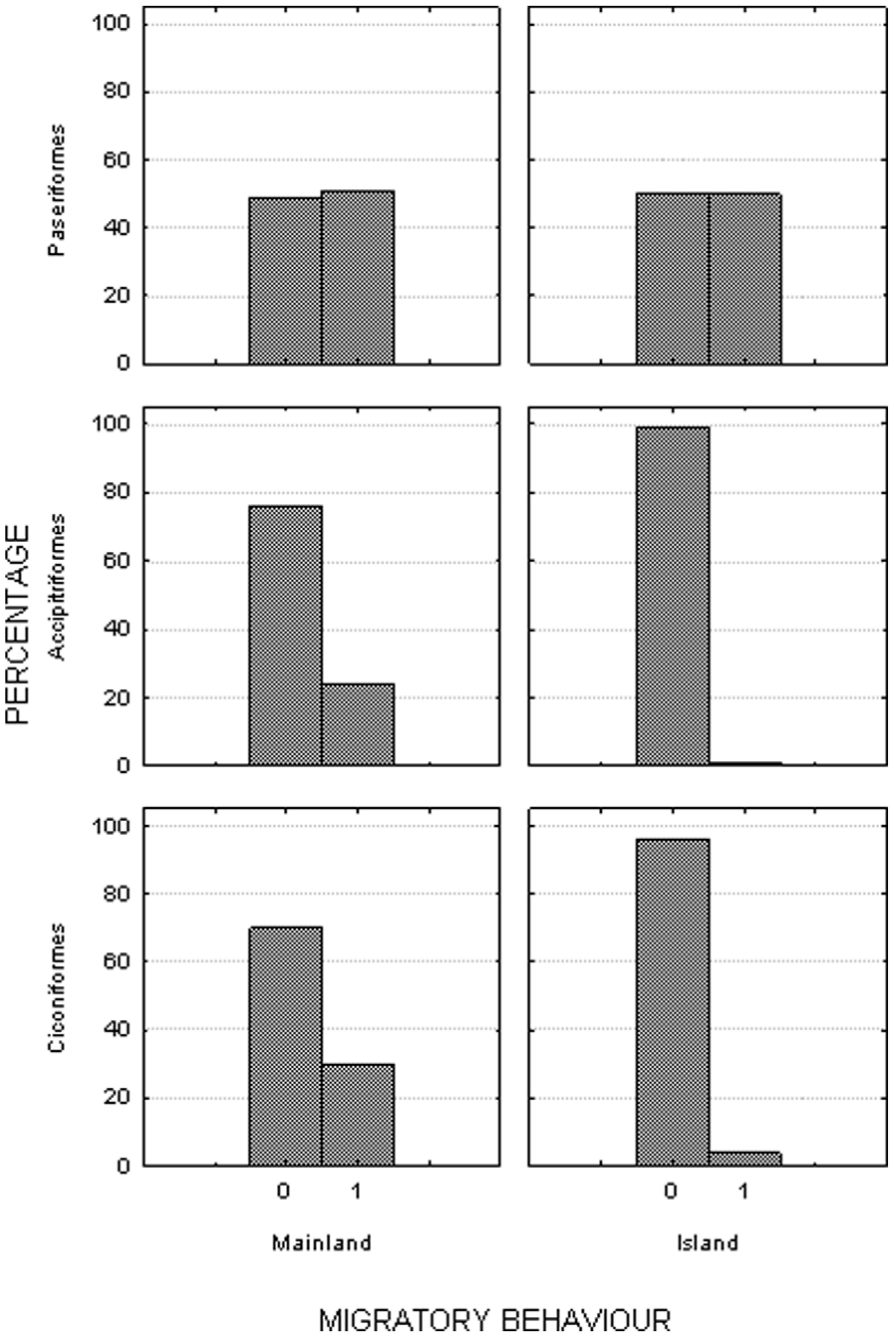
Percentage of species of passerines, raptors and Ciconiiformes species with migratory habits on island versus mainland (sedentary species  =  0, migratory species  =  1). Passerines do not changes of migratory habits between island and the mainland. In contrast, raptors, storks and herons changed to all-but-entirely sedentary on islands.

There are 314 species of raptors worldwide [22]. Globally, 77 (25%) of all raptors are mainly migratory, and 237 (75%) are mainly sedentary. Only 165 of the 314 species of raptors occurred on islands (53% of all species). Significant differences were found between the proportion of migratory and sedentary species of raptors on islands versus the mainland (Fisher's exact test, P<0.001). Only three (2%) of the 165 species with insular populations are migratory on islands. In addition, 20 species with both continental and insular populations are migratory on the nearest continental population and sedentary on islands (12% of species on islands). None of the 142 sedentary continental species of raptors is known to be migratory on islands (McNemar test  = 131.34, P<0.001, Figure 1). Sedentary behavior on islands seems stable with no case of a species being sedentary in some islands and migratory in others.

There are 113 species of Ciconiiformes worldwide, 34 (30%) of which are migratory and 79 (70%) of which are sedentary. Fifty-two (46% of all species) of these species occur on islands. Of them, only two (4%) are migratory on islands, the remaining 50 species are sedentary. Significant differences were found between the proportion of migratory and sedentary species of Ciconiiformes on islands versus the mainland (Fisher's exact test P<0.001). In addition, six species with both continental and insular populations are migratory on the mainland and sedentary on islands (12% of species on islands), whereas none of 79 sedentary continental species is known to be migratory on any island (McNemar test =  6.13, P<0.01, Figure 1). Sedentary behavior on islands seems stable with no case of a species been sedentary on some islands and migratory in others.

Among the 136 species of passerines (belonging to Corvidae, Sturnidae, Passeridae, Estrildidae, Vireonoidae, Fringillidae, Parulidae, Thraupidae, Emberizidae, Cardinalidae and Icteridae families) whose migratory tendencies were analyzed, 69 (51%) were migratory and 67 (49%) were sedentary. Ninety-two (68% of all species) occurred on islands. Of these, 46 (50%) were migratory and 46 (50%) were sedentary. No differences were found between the proportion of migratory and sedentary species of passerines on islands versus the mainland (Fisher exact test p = 0.788, Figure 1). Among 92 species of passerines that had both continental and insular populations, none was migratory on the mainland and sedentary on islands, and none was sedentary on the mainland and migratory on islands. Overall, significantly more passerines than Ciconiiformes and Accipitriformes were migratory on islands (Fisher exact test P<0.001, Figure 1).

**Figure 2:**
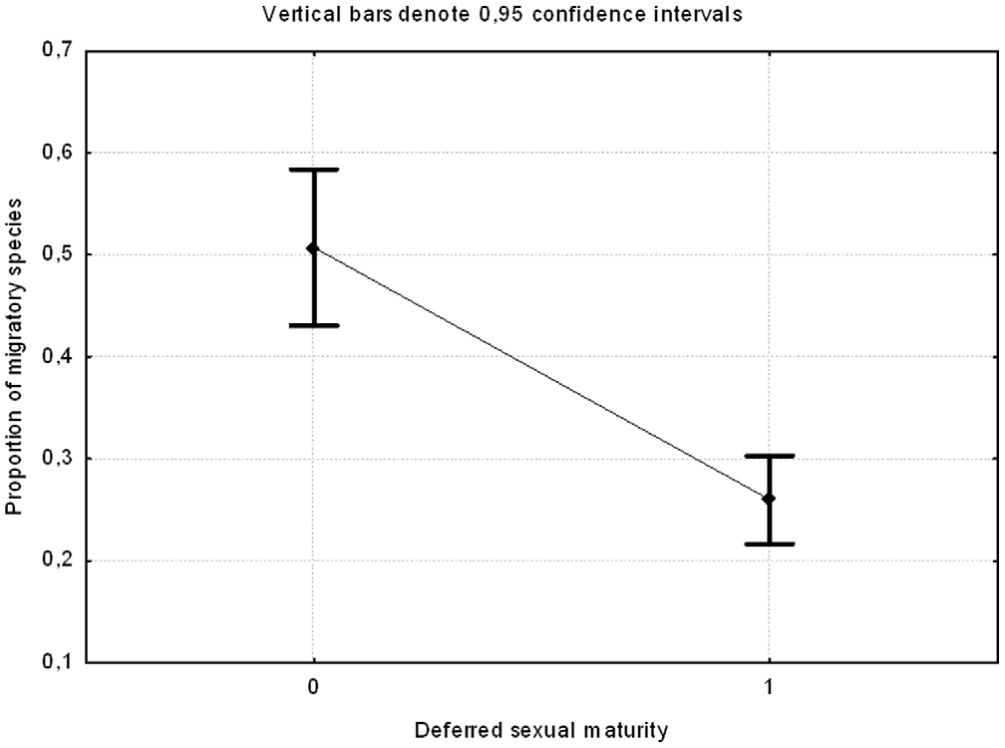
GLM of proportion of migratory species according the existence of deferred sexual maturity. Accipitriformes and Ciconiiformes showed lower proportions of migratory species than did passerines.

The proportion of migratory species for the three orders was significantly related to the presence or absence of deferred sexual maturity (GLM, binomial distribution and logit link, Wald statistic = 27.81, df = 1, P<0.001, Figure 2). Those orders with deferred sexual maturity (i.e. Accipitriformes and Ciconiiformes) showed a lower proportion of migratory species (26%) than passerines (51%, Figure 2).

Sedentary behavior on islands was related to the presence of deferred sexual maturity, with those species showing deferred sexual maturity being largely sedentary on islands (GLM binomial distribution and logit link, Wald statistic = 7.358, df = 1, P = 0.006), but not with taxonomical order (GLM binomial distribution and logit link, Wald statistic = 1.787, df = 2, P = 0.181).

We obtained wing loading data for 409 species belonging to Accipitriformes (254), Passeriformes (131) and Ciconiiformes (24). We found significant differences among taxonomical orders in wing loading, (ANOVA log wind loading; F_2, 406_  =  125.63, P<0.001), with higher values in Ciconiiformes and Accipitriformes than in Passeriformes. Analyzing only Accipitriformes, the order with a greater number of species with data, wing loading was not related to a change from migratory to sedentary behavior on islands (GLM binomial distribution and logit link, Wald statistic = 0.061, df = 1, P = 0.804). Also, considering only those species belonging to the three orders present on islands, wing loading showed no relationship with change in behavior from migratory in the mainland to sedentary in the island (GLM binomial distribution and logit link, Wald statistic = 0.161, P = 0.162).

No differences in nearest distance to the continent were found among birds of the three taxonomical orders living on islands (ANOVA log of nearest distance to mainland, F_2, 242_ = 0.530, P = 0.589). Frequency of change from migratory to sedentary behavior on islands was not related to distance to the nearest continent (GLM binomial distribution and logit link, Wald statistic = 2.117, df = 1, P = 0.145).

Results of GLM model building, using binomial distribution and logit link, with change in migratory behavior to sedentary behavior on islands (in the same species from migratory populations on the continent to sedentary populations on the nearest island) as response variable and delayed reproduction and taxonomic order as explanatory variables, and wing load as covariate are show in Table 1. The best explanatory model according to Akaike Information Criterion (AIC) was the one including only deferred sexual maturity. Taxonomic order was not included in the most explicatory model. Consequently, individuals coming from a migratory population with deferred sexual maturity that settled on islands have a significant probability to change from migratory to non-migratory behavior.

**Table 1:** Modeling results of GLM with binomial distributions and logit link function with change from migratory to sedentary behavior on islands as response variable and delayed reproduction (D. maturity) and taxonomical order as explanatory variables, and wing load as covariate.

Step	Var. 1	Var.2	Var.3	AIC	Δi	*wi*
1	D. maturity			123.429	0	0.5585
2	Wing load	D. maturity		125.425	1.99	0.2059
3	Order			127.088	3.65	0.0899
4	Order	D. maturity		127.640	4.20	0.0682
5	Wing load	Order		128.420	4.98	0.0462
6	Wing load	Order	D. maturity	129.020	5.76	0.0313
7	Wing load			147.286	24.03	0.0000

We use the best subset approach and the AIC value (AIC =  Akaike Information Criterion).The best-fitted model was the one including only deferred maturity according to AIC weight value (*wi*). Migratory birds with deferred maturity (i.e. delayed return) change to sedentary on islands.

**Figure 3:**
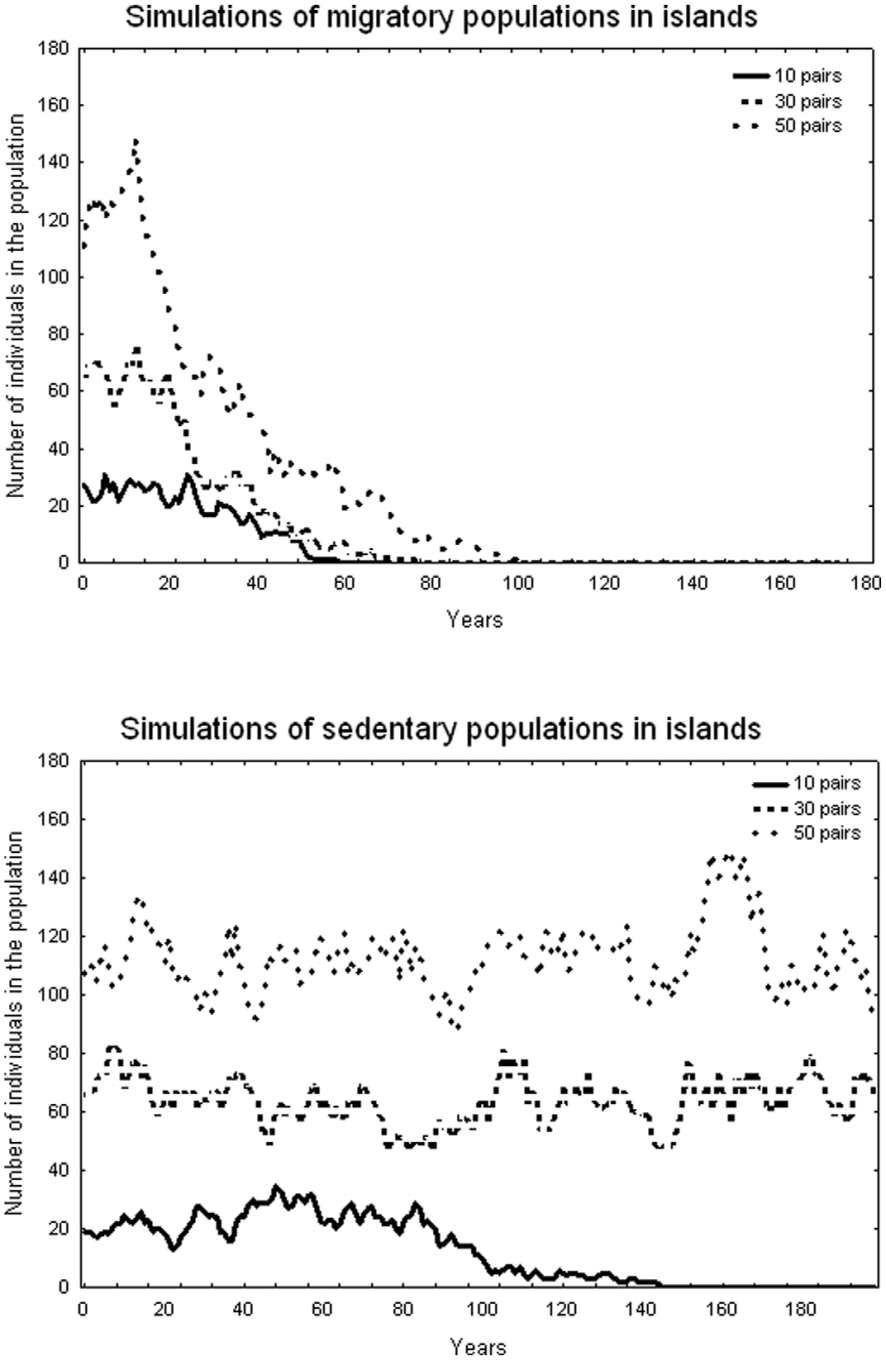
Results of selected individual simulations that was closest to the mean of 200 replicates of different combinations of carrying capacity and migratory habits in species with deferred sexual maturity. Figure a (high) show the trajectories of migratory populations of different sizes in island. Figure b (low) show the same situation but with sedentary populations.

Results of selected individual simulations that were closest to the mean of 200 replicates of different combinations of carrying capacity and migratory habits are shown in Figure 3. The time that a population persists is clearly affected both by population size and by movement ecology. Persistence increased as breeding population size increased, and was higher in simulations with an island able to host 50 breeding pairs. Time to extinction was significantly longer for simulated sedentary populations than for simulated migratory populations (stratified proportional hazard (Cox) regression for censored data n = 1,200, uncensored data 62.83%, log-likelihood of final solution  =  −3,573.60, Null model  =  −4,147.21, χ^2^ = 1,147.230, df = 1, P<0.001).

We analyzed breeding population fluctuations (i.e., breeders present annually, expressed as a percentage of the upper population size) during the first 50 years of simulation in those simulations that survived for at least 100 years. Mean percentage of pairs present during the first 50 years was affected by sedentary behavior but not by upper breeding population size (2-way ANOVA; Sedentary behavior, F_1, 2_ = 107.59, P = 0.009; Island size, F_2, 2_ = 7.77, P = 0.114). Percent of population saturation was higher in sedentary than in migratory populations.

## Discussion

To our knowledge, the fact that long-lived birds with deferred sexual maturity almost always shift from a migratory to sedentary lifestyle once they have established themselves on islands, has not been described previously. This phenomenon is in contrast to what occurs in passerines, a group in which age at first breeding is rarely deferred and that regularly exhibit migratory behavior on islands.

One prediction of our hypothesis is that sedentary populations of species with deferred maturity will have higher percentages of breeders in non-adult plumage than will migratory populations. As many as 12% of Egyptian Vultures in a 25-pair breeding population on the Canary Islands breed in sub-adult plumage [23] compared with none among the 1,500-pair migratory population breeding in mainland Spain. Thus, the average age of first reproduction on the Canary Islands was 4–5 years, compared with 7–8 years on the mainland [21]. As many as 35% of sedentary Spanish Imperial Eagles breed in sub-adult plumage (i.e., <5 years of age) in some years in certain locations, and the average appears to be 10% for the 200-pair population as a whole [8]. By comparison, there is only two known cases among 160 pairs of an individual breeding in sub-adult plumage (<<1%) in the closely related but migratory Eastern Imperial eagle. In both examples, the difference between migratory and sedentary populations or species was significant (percentage test, P<0.001).

The exact behavioral shifts that produce these differences have yet to be studied in detail. That said, while the recently fledged young of sedentary Broad-winged Hawks (*Buteo playtypterus*) breeding on the West Indies on the island of Puerto Rico do not disperse from their nest sites and maintain home ranges that overlap with those of breeding adults [24], the young of continental populations begin migrating thousands of miles south toward wintering areas in Central and South America within two months of fledgling [25], suggesting that such shifts include changes in the extent of juvenile dispersal.

Our investigations indicate that only three species of raptors and two species of herons are migratory on islands. They are the Eleonora's Falcon (*Falco eleonorae*), an exceptional “marine raptor” that lives mainly on small, continental islands; the Rough-legged Buzzard (*Buteo lagopus*), and the Montagu's Harrier (*Circus pygargus*) among the raptors and the Madagascar Pond Heron (*Ardeola idea*) and the Japanese Night Heron (*Gorsachius goisagi*) among herons. The absence of delayed return for these species could be an explanation. Unfortunately, there are no data regarding this possibility. Assessing the extent to which young birds breed in these species is needed.

Traditionally, the idea that many land-based birds, including raptors and Ciconiiformes [17], [26], avoid flying over large bodies of water because of the risks involved in doing so, has been used either directly or indirectly to explain the paucity of overwater dispersal of birds from islands [27]. That said, this does not explain why, among the passerines we studied (body masses <20 g–1,500 g), none appeared to change their migratory habits upon establishing themselves on islands, whereas almost all of the migratory raptors, storks and herons that we studied did. Indeed, whereas 87% of the migratory raptors and 75% of migratory Ciconiiformes we investigated became sedentary on islands, none of the passerines we investigated did so. The absence of any relationship between wing loading and changes in migratory behavior on islands seems to support the idea that differences in flight abilities do not explain this difference.

We believe that density-dependent variation in the age of first breeding does explain this difference. Variation in the age at first breeding appears to be a critical aspect of the viability of small isolated populations in general, and for small insular populations in particular [10]–[12]. The buffering effect of this variation allows insular populations to remain closer to their carrying capacities more often, which, in turn, affects population persistence significantly. Differences between mainland and island populations in the proportion of young breeders strongly support this interpretation.

### Conclusions

The probable evolutionary consequences of this shift in migratory behavior remain to be seen. Nevertheless, if migratory populations became sedentary when colonizing islands, it is clear that genetic divergence from the original migratory population would be favored. In isolated sedentary populations, recruitment will be mainly on the offspring of the same breeding population. That is true even if the island is part of the mainland population's migration corridor. The probability that vacancies in breeding population are occupied by an individual depends upon the abilities of potential individuals to detect it. This ability should be greater in sedentary floaters than in migratory floaters, assuming vacancies occur year round.

Several endemic species of raptors and herons now living on islands appear to be descendents of migratory mainland ancestor [22], [28]. Examples include the continental migratory Lesser kestrel (*Falco naumanni*) and Amur falcon (*F. amurensis*) and the group of insular sedentary species including the Malagasy spotted kestrel (*F. newtoni*), the Mauritius kestrel (*F. punctatus*), the Seychelles kestrel (*F. araea*) and the Moluccan kestrel (*F. moluccensis*).; the meta species group of serpent-eagles in the genus *Spilornis* in Malaysia; and the continental migratory Cooper's hawk (*Accipiter cooperii*) and the insular sedentary Gundlach's hawk (*A. gundlachi*) of Cuba [22]. This suggests that increased genetic divergence due to shift to sedentary behavior on islands may play an important role in bird speciation [17].

Our analysis suggests that the shift from migratory to sedentary behavior must happen quickly for small populations to survive due to the inherent risk of extinction for migratory small populations of birds with deferred sexual maturity. As such, migratory behavior in birds with deferred sexual maturity appears as an important constraint affecting viability of populations on islands. Probably the same argument could be used for small continental populations. As long-lived migratory species may not be able to survive for long periods in small populations, colonization of remote areas, including islands, is easier if the birds involved become sedentary. The existence of pairs of migratory-sedentary species in raptors, storks and herons should be re-analyzed with this taken into account.

Our results also support the idea of inherent phenotypic flexibility in migratory behavior in individuals facing new circumstances on islands as well as a possible rapid-genetic-selection process within island populations [20], [17]. One way to determine the extent to which each of these two processes contributes to enhanced sedentary behavior on island populations of birds with ancestral deferred reproduction would be to conduct an experiment of translocation of nestlings from sedentary islands population to nests of migratory continental populations of the same species and vice versa.

## Materials and Methods

We analyzed the migratory behavior of Accipitriformes and Ciconiiformes, two taxa with frequent deferred sexual maturity and delayed return migration. We then compared their behavior to those of small passerines Passeriformes, a group of birds without deferred maturity. We considered all species of Accipitriformes and Ciconiiformes and passerines representing 11 widespread families (i.e., Corvidae, Sturnidae, Passeridae, Estrildidae, Vireonoidae, Fringillidae, Parulidae, Thraupidae, Emberizidae, Cardinalidae and Icteridae). We examined the occurrence of migratory behavior on islands (i.e., small isolated populations) in both groups. We considered islands to be those that received their fauna via sea crossings and not land bridges [cf. 7].

We used the most recent literature reviews for raptors [17], [22], ciconiiformes and passerines [18], [29] to determine the presence of migratory populations on small islands. Where species occurred both on islands and on the mainland, we paired insular populations with their nearest continental populations at the same latitude, and then compared the extent of migration behaviour within and between each member of the pair. Data from Egyptian Vultures (*Neophron percnopterus*), Spanish Imperial Eagles (*Aquila adalberti*), and Eastern Imperial Eagles (*Aquila heliaca*) were used to test differences in the proportion of birds breeding in immature plumage in sedentary versus migratory populations.

In order to analyze possible effects of flight abilities in migratory behavior on islands versus mainland, we estimated wing loading for those species with data using published data [17], [18], [22], [29]. We used mean weight divided by 2*wing length*tail length. When data of different subspecies were available, the nominal subspecies was tested. Additionally, to analyze the possible effect of distance from mainland to the island on migratory behavior, we measured the distance to the nearest continent for each species present on islands.

To compare the frequency of migratory-sedentary species in and between the three orders, we used non-parametric analyses. We used Fisher's exact tests (two-tailed) to compare proportions between mainland and island of migratory versus sedentary species belonging to the same order. To compare number of species that change from migratory on mainland to sedentary behavior on island versus number of species that changes from sedentary behavior on the mainland to migratory on islands we used McNemar tests (designed to compare changes among dependent samples).

Generalized Linear Models (GLM) with binomial distribution and logit link function were used to analyze the effect of delayed reproduction and taxonomy on both migratory behavior on islands and frequency of change from migratory to sedentary on islands. In the first case, the response variable was the existence or not of migratory behavior on islands, and explanatory variables were the presence or absence of delayed reproduction and taxonomy (i.e. order). We also analyzed probability of change in migratory behavior on islands in relation to nearest continental population of the same species. For this purpose, we conducted a GLM with “change to sedentary on island” as response variable with binomial distribution, and delayed reproduction and taxonomical order as explanatory variables and wing load as covariate. Other GLM analyses were also conducted, for example to test for the effect of wing loading in migratory behavior or the effect of distance to the continent in the probability of change from migratory to sedentary behavior.

We used individual-based simulation analyses to examine extirpation probabilities for sedentary versus migratory populations living on islands. A typical large-raptor population case-study was simulated using data from the Spanish Imperial Eagle (*Aquila adalberti*) that included age structure, fecundity, mortality and density-dependent relationships revealed in independent analyses [10]–[12], [29]. To analyze differences between sedentary and migratory behavior in small island populations of this eagle we conducted 200 simulations of each combination of input variables across a 200-year test period, with different combinations of carrying capacities, 10, 30 and 50 pairs). We simulated two different scenarios. In sedentary populations, early reproduction was permitted from 3 years of age, even though adult plumage was set to be acquired at 5 years, when vacancies exist and all adults are already paired [10]. Thus, age at first reproduction in sedentary populations was incorporated as a density-dependent variable. In migratory populations, reproduction was permitted only at and beyond a minimum age of 5 years (i.e., the age at which adult plumage is acquired) to model the assumption that in migratory populations birds in juvenile and sub-adult plumages do not return to their insular breeding ground and attempt to breed due to delayed return. A stochastic individual-based simulation using Delphi-5 has been designed to model the dynamics of both types of populations, sedentary versus migratory populations. The model was based on previous published models [10], [12], and included environmental and demographic stochasticity in mortality rates and fecundity. Density-dependent fecundity is modeled according real field data 30. For each case, we display as representative the individual simulation that was the closest to the mean for that series of 200 replicates. Finally, a proportional hazard (Cox) regression for censored data was conducted to analyze the effect of density-dependent age of first breeding on time to extinction, stratified by the three carrying capacities considered. Statistical significance was set at P<0.05. All analyses were carried out using STATISTICA.7 package.
